# EFL Teachers’ Immediacy and Professional Commitment on Students’ Boredom: A Review of Literature

**DOI:** 10.3389/fpsyg.2021.808311

**Published:** 2022-02-07

**Authors:** Wen Qin

**Affiliations:** School of Education, Anyang Normal University, Anyang, China

**Keywords:** teacher immediacy, teacher-students interaction, positive psychology, professional commitment, boredom

## Abstract

Boredom is a psychological phenomenon that is defined as a state of hatred or incompatibility with any kind of repetitive experience in situations where liberation from instability is not possible and has several consequences. Boredom is one of the important causes of decreased motivation in EFL learners, and it is necessary to identify the factors affecting it. Therefore, this literature review addresses the state of boredom in relationship with teachers’ immediacy and professional commitment. Reviewing the literature has revealed that while teachers’ immediacy and professional commitments affect EFL learners’ boredom, other factors such as individual differences and environmental factors are in action. However, many studies confirmed that teachers play the most important role in decreasing or increasing learners’ state of boredom. The findings are significant for teacher educators to design more appropriate teacher education programs.

## Introduction

The need for coexistence and human relationships in human life is important. In the shadow of human relations, the human psyche is softened and its talent blossoms (Rothes et al., 2017; Lazarides et al., 2018). Education is a change in behavior, and educational environments are a place of change and transformation (Malik et al., 2018). The most important factor in the development of scientific mental development in a society is the educational system. In this system, from the beginning to the last specialized course, scientific ethics, social ethics, and how to deal with issues and problems are taught. Teaching is one of the main elements of the education process that plays an effective role in the efficiency of the educational system (Ouellette-Schramm, 2019; Zawodniak et al., 2021). In educational systems, many people are employed and their activities directly or indirectly affect students’ education, but in the meantime, the role of teachers is much more prominent than other people because students spend most of their time with them. Other factors also provide the basis for the activities of teachers; so many graduates of educational systems owe their personality formation to their teachers (Dewaele et al., 2018; Derakhshan et al., 2020a).

What makes a teacher’s job difficult is the creation of a learning environment and finding appropriate ways to attract language learners. It must be acknowledged that language learners’ talents and their learning styles are different (Pishghadam et al., 2020; Inan-Kaya and Rubie-Davies, 2021). Some language learners are very talented and learn quickly, and some need more work and practice. What matters is how the teacher fits into the language learners’ minds. The power to control and take over the classroom is the art of teachers (Rigby and Ryan, 2018). While teaching methods and teaching materials play significant roles in the language teaching process, affective factors are among the main challenges of language classrooms (Li et al., 2018; Talebzadeh et al., 2020; Alavi et al., 2021; Wang Y. L. et al., 2021). Recently, students’ boredom has received special attention in language teaching research studies. Researchers have used the Control-Value Theory (CVT) of achievement emotions in defining and researching positive or negative emotions especially students’ boredom in EFL contexts (Pekrun, 2006). This theory provided theoretical bases for language researchers in educational psychology. Based on this theory, language learners’ emotions have environmental and individual determinants. Among numerous existing factors in any language teaching contexts, many studies asserted that in creating a friendly atmosphere in language classrooms and avoiding students’ boredom teachers need to balance among teachers’ immediacy and professional commitment (Khajavy et al., 2018; Yang and Yin, 2021).

Despite the enormous psychological research on emotions since the early 1980s (Rigby and Ryan, 2018), little of them has informed research on teachers’ immediacy and professional commitment. Researchers know surprisingly little about the role of emotions in learning to teach, how teachers’ immediacy and professional commitment relate to their students’ boredom. Researchers also know little about how teachers regulate their emotions, the relationship between teachers’ emotions and learning. In this article, the literature related to teachers’ immediacy and professional commitment is reviewed. The article begins with the empirical literature that focuses on the range of positive and negative emotions that teachers experience is summarized and critiqued. Next is an analysis of the various ways in which teachers’ immediacy and professional commitment may influence their students’ boredom. Finally, several future directions for research are suggested.

## The Study

In order to encompass various aspects of the topic, this literature review begins with a question, “What does the educational research say about teachers’ immediacy and professional commitment toward language learners’ boredom?” The purpose and objectives of this review are (a) to discuss the findings of studies that focus on this topic and (b) to provide important information to teachers, teacher educators, school principals, and policymakers on how to enhance teachers’ beliefs on improving learning opportunities and quality of education for English language learners. The following research question guided the study:

What does the educational research say about teachers’ immediacy and professional commitment toward language learners’ boredom?

### Teachers’ Immediacy

Teacher-student interaction is one of the significant elements of the language teaching process. It seems that another variable affecting teachers’ professional commitment as well as students’ performance and satisfaction in the classroom is close communication and two-way conversation between teachers and students (Cakir, 2015; Xie and Derakhshan, 2021). Two-way conversation refers to bilateral dialogue, a two-way and a reciprocal process in which all participants are effective in creating the process and receiving messages (Fallah, 2014). Teachers and principals are asked to put themselves in students’ position, which reduces the separation and boredom of students in the learning environment during intensive interaction (Keller et al., 2016). This empathetic collaboration leads to a deeper understanding of the processes and consequences of education. Finally, a form of individual activity and social interaction takes place between members of the educational environment and a common understanding is created about students, learning and the educational process (Putwain et al., 2018). The communication process is a continuous and advanced exchange in which participants become fully aware of their teachers and the others in the educational community (Mellati and Khademi, 2018; Derakhshan et al., 2019; Wang and Guan, 2020; Xie et al., 2021). A two-way communication is a fundamental and reciprocal process that moves toward discovering and understanding new information by listening and talking.

Numerous factors attributed to teachers’ emotional arousal affect the teachers’ immediacy and subsequently influence the quality of language teaching and students’ boredom. One of the factors that play a crucial role in students’ boredom is teachers’ immediacy. Since this factor can mediate among students’ academic motivation, students’ self-regulation, and classroom culture (Hiver et al., 2021), motivational paucity also plays an indispensable role in investigating the reason for language learners’ boredom and dropout. It means language learners’ emotional status do not arise as a result of a simple cause-and-effect relationship, but rather appear to be the result of regular interactions of teachers and learners in their learning environment with their specific culture (Derakhshan, 2021). Therefore, it can be argued that language learners’ emotions is influenced by their teachers’ immediacy and the messages they perceive from the language learning context (Linnenbrink-Garcia and Pekrun, 2011). Teachers’ immediacy might promote healthy socio-emotional development and also lead to adaptation in language learning environments (Pishghadam et al., 2019). Despite its obvious scientific aspect, this relationship is not limited to the transfer of scientific and technical teachings from a teacher to language learners (Li et al., 2018; Derakhshan et al., 2020a). Language learners also perceive behavioral culture, personal character, social perspective, and teaching style. Unlike mechanical relationships, the teachers’ immediacy in the classroom is a complex human relationship in which various factors such as abilities, personality, and family circumstances are in interaction (Wang and Guan, 2020; Xie and Derakhshan, 2021). Effective communication has well-known principles and techniques that EFL teachers need to be familiar with in creating the most adequate language learning environment (Rothes et al., 2017).

However, language teachers have some challenges such as different levels of parental involvement, decomposing facilities, the risk of violence, and diverse learners in their teaching environments (Pennington and Richards, 2016). Teacher immediacy might influence every aspect of the language teaching process, from language policy to a specific teaching method; therefore, researchers have paid special attention to the relationships between teacher immediacy and their professional commitments (Derakhshan et al., 2020b; Wang G. et al., 2021).

### Professional Commitment

Today, educational environments need more and more hard-working and committed human resources, and the realization of the goals of education, more than anything else, should be considered as the effort of teachers (Vogel-Walcutt et al., 2012; Mellati et al., 2013; Wang G. et al., 2021). Among these, one of the most important factors and areas studied is the organizational or professional commitment of teachers. Professional commitment is considered as one of the most important determinants of EFL teachers’ work behaviors and is an issue that has attracted the attention of many researchers and managers in educational settings (Tze et al., 2013; Derakhshan et al., 2020a). Professional commitment is defined as a sense of identity and dependence on a particular job and profession and on the desire and interest to work in a profession. In addition, professional commitment is defined from the perspective of social identity theory (Daniels et al., 2015). This theory holds that individuals categorize themselves into different social classes and thereby define themselves in terms of membership in a particular entity. That is, people are interested in being in a group or a group of society (Daschmann et al., 2014). Professional commitment is defined as identification with a profession and attachment to the profession, and is described by three characteristics: belief and acceptance of professional goals and values, willingness to work hard based on teachers’ own beliefs, and willingness to keep members in that profession (Mellati et al., 2018; Putwain et al., 2018; Fathi et al., 2021).

Research in the field of organizational behavior has shown that professional commitment consists of three dimensions, each of which develops in a different way and leads to different results (Moskowitz and Dewaele, 2021; Nakamura et al., 2021). These three dimensions are: Active professional commitments: This type of commitment arises through positive professional experiences or the development of professional skills; Continuance professional commitment: When people feel they have to stay in their profession because of a build-up of capital or a lack of comparable options; and Normative professional commitment: When people feel compelled to stay in their profession through a sense of compulsion (Daschmann et al., 2014; Mellati et al., 2015; Khajavy et al., 2018; Lazarides et al., 2018; Morris, 2019). It seems that two categories of factors affect teachers’ professional commitment: individual factors and organizational factors. Individual factors include variables that are related to individual characteristics and personal life. Organizational factors include characteristics that affect the individual through the organization (Lazarides et al., 2019; Derakhshan et al., 2020b). One of these individual factors is language learners’ boredom that has received special attention.

### Students’ Boredom

Boredom is defined as a state of hatred or incompatibility with any repetitive experience such as daily work or dealing with dull and boring people and excessive restlessness in situations where it is not possible to get rid of monotony (Daniels et al., 2015; Dewaele and Li, 2021). It is phenomenologically related to similar states such as apathy and indifference, lack of pleasure and depression. Boredom is a psychological phenomenon that has several complications (Kruk, 2019; Finkielsztein, 2020). Decreases in professional and academic performance, increased risk of mental disorders such as anxiety and depression, hopelessness, feelings of loneliness and aimlessness in life and, most importantly, increased risk of leaving the educational environment are among the negative consequences of the phenomenon of boredom. Boredom in general is an unpleasant emotional state in which learners are not interested in what they are doing and cannot focus on it (Nett et al., 2010; Pawlak et al., 2020, 2021).

According to previous studies, various conditions lead to boredom. Some of the predictors of boredom that have been confirmed in various studies are: *Academic predictors*: include inappropriate learning environment and hostile relationship between teachers and students. *Psychological predictors*: the most important psychological predictors are boredom, meaninglessness and aimlessness in life. *Cognitive predictors*: difficulty maintaining attention and cognitive skills. *Personality predictors*: include motivational orientation and sensory search. Academic and psychological predictors highlighted the importance of teacher-students interactions in predicting language learners’ boredom and their language achievements (Sulea et al., 2015; Derakhshan et al., 2021a,b).

Numerous studies have been conducted to explore the relationships between teachers’ immediacy, teachers’ commitments, and students’ boredom (Linnenbrink-Garcia and Pekrun, 2011; Keller et al., 2016; Dewaele et al., 2018; Khajavy et al., 2018; Fathi et al., 2021). Cakir (2015) investigated the impact of teachers’ immediacy and students’ boredom. The results of this study that was conducted in Turkish context indicate that teacher-students interaction is a strong predictor of learners’ empowerment in educational contexts. The findings of this study suggested that training courses, seminars, and conferences can be organized for language teachers to enhance their knowledge of teacher immediacy and different strategies they can apply in their classrooms to increase teacher-students interaction in language contexts. Fallah (2014) examined the connection between teacher-students interactions and their willingness to communicate. The results of this study conducted in Iranian EFL context revealed that there is a direct and positive relationship between students’ motivation, self-confidence, and willingness to communicate and teachers’ immediacy. In addition, the findings showed that teachers’ immediacy has a direct and negative impact on students’ shyness. Malik et al. (2018) focused on professional commitment and found that professional commitment was contributed to educational policies and have a significant impact of students’ knowledge and achievements. Sulea et al. (2015) explored the relationship between boredom, burnout, and Engagement and personality traits among EFL students and found that individual personality traits predict students’ boredom in language classrooms. Dewaele and Li (2021) investigated the role of teachers’ enthusiasm on Chinese EFL learners’ boredom. After collecting the required data from 2002 Chinese EFL learners they found that there is a direct and positive link between teachers’ enthusiasm and learners’ emotions. They argued that learners’ enjoyment, boredom, and engagement can be affected and determined by teachers’ enthusiasm. Xie et al. (2021) investigated contributing factors in academic boredom. They emphasized on the role of teachers and their classroom interactions as a major source of determining learners’ boredom. Zawodniak et al. (2021) found that teachers’ educational tasks are the major source of learners’ boredom in language classrooms.

In addition, Derakhshan et al. (2021b) explored the sources of boredom in online classes as a new mode of instruction. They found that teachers’ specific activities or educational tasks in their classrooms affect EFL learners’ boredom. They argued that teachers’ immediacy can be an undeniable solution to reduce EFL learners’ boredom and increase their engagement in language classrooms. In a same vein, Nakamura et al. (2021) highlighted the significant role of teachers and their activities in language classrooms in decreasing or increasing learners’ boredom in language teaching contexts. In a similar study, Yang and Yin (2021) found that teachers’ immediacy and teacher-student interactions in the classrooms reduce teacher-student distance and increase their speaking and writing production and their willingness to communicate. In another study, Wang G. et al. (2021) highlighted the role of teacher professional commitment in creating effective teacher-student interactions in the class and decreasing learners’ boredom. After reviewing 47 empirical studies, they focused on the moderating role of teacher professional commitment in determining learners’ boredom. In contrast, Inan-Kaya and Rubie-Davies (2021) argued that in the same situations learners show completely different behaviors. They believed that individual differences not teachers’ interaction or teacher immediacy lead to learners’ boredom.

While many studies contributed the notion of students’ boredom in language classrooms to the inadequate teacher-students interaction, Pawlak et al. (2020) suggested that monotony, repetitiveness, and disengagement as the first factor and lack of satisfaction and challenge as the second factor play key roles in predicting language learners’ boredom. In addition, Tze et al. (2013) in their studies on students’ boredom argued that this notion is a context-bound and culture-bound notion. They stated that the way Chinese students cope with boredom is completely different from the way Canadian students do; therefore, more studies in different contexts are required to shed light on latent aspects of this multidimensional notion.

## Discussion

Reviewing the literature has revealed that there is a significant relationship between language learners’ boredom and environmental factors such as university factors, home factors and residence factors. The studies have also shown that there is no significant relationship between boredom and social factors, gender, indigenousness, marital status, employment, and physical activity. Regarding the role of mediation, two-way communication, and professional commitment of teachers, it should be noted that the process of two-way communication with its constructive features both directly increases the professional commitment of teachers and indirectly increases the relationship between teachers and language learners (Lazarides et al., 2019). In addition, a two-way communication that both parties are aware of each other’s views and opinions is very valuable in reducing misunderstandings and misinterpretations and learners’ boredom. In the process of two-way and reciprocal communication, if both parties have the right to comment and express their opinions, the thoughts come together and a kind of empathy and effective cooperation between teachers and learners is created. This two-way communication can lead to the integration and harmony of the learning environment (Nakamura et al., 2021).

Many studies highlighted the crucial role of teachers in determining appropriate educational tasks and the way they conduct those tasks in the class. They argued that the teachers’ tone of voice influences learners’ reactions and boredom (Morris, 2019; Pawlak et al., 2020). The teachers need to know when to raise and lower their voice to get the students’ attention. If the students do not feel comfortable in the classroom, the teachers’ efforts will not applicable. The way teachers behave, speak and even look has special impact on language learners’ emotions and their boredom. If the atmosphere of anxiety and solidarity prevails in the class or there is no control over it, the class becomes boring; it becomes chaotic; and eventually leads to not paying attention to what is presented (Li et al., 2018; Derakhshan et al., 2019).

Malik et al. (2018) argued that disobedient and playful language learners can be controlled by giving them various responsibilities such as homework control, attendance, distributing and collecting exam papers, and liaising with the school office. The teachers’ attention to language learners has a significant effect on their satisfaction. Even a simple glance or encouragement or criticism in private and far from being presented to the language learners can be very effective. Derakhshan et al. (2021a) believed that the teachers’ attention to students’ issues and personal characteristics is also one of the things that encourage them to be more effective in the classroom. The way a teacher treats a shy and reckless language learner is different, and the way encouragement and punishment are applied is different.

The basis of change is love and affection that is achieved in human relationships. How can the principal communicate with the teacher, the principal with the students, and the principal with the parents? How can they convey their feelings and emotions to them and cause change? One of the important factors that have an undeniable effect on human relations in educational environments is the conditions and atmosphere prevailing in the educational environment and teachers, as coordinators, have more responsibility in creating a friendly atmosphere.

Nett et al. (2010) stated that the emotional relationship between teachers and student, the inadequacy of the educational environment and the students’ lack of comfort affect their learning. Teachers should be careful to not create a sense of punishment in the classes. This means that if the students are mischievous, the teacher must have the patience to accompany them. But if, on the contrary, Pawlak et al. (2020) and Pishghadam et al. (2020) argued that they are always punished, in fact, they have created a sense of punishment in them. This prevents language learners from learning, and teachers think that they are failing, which is not the case.

Zawodniak et al. (2021) investigated the role of teacher in language assessment and asserted that teachers have to adapt the conditions, facilities, and tests to the students. If there is a problem in the teaching and learning process, we should not blame the student, but we should review the situation. Putwain et al. (2018) stated that all factors inside and outside the education system must help to achieve this. The community should provide appropriate contexts in line with what students learn in school, for example, if they learn about health, morals and social issues at school, they should practice them in community. Society should be a reflection of what students have learned. If the educational environment and the society in which the students live are not complementary and contradictory, there will be a gap that will lead to boredom and academic failure of students. Similarly, Sulea et al. (2015) and Rothes et al. (2017) argued that the relationship between student and teacher should also be highly emphasized, in a way that their interaction is the most important element in teaching and giving personality to the student. If this interaction is emotional, scientific, intimate and strong, the students’ trust is gained and consequently the quality of education is improved. Therefore, human resources play a key role in the quality of training. Teacher education programs need to move toward courses in which teachers gain professional competencies and skills and work with motivation and interest alongside expertise.

In contrast, Vogel-Walcutt et al. (2012) and Talebzadeh et al. (2020) highlighted the crucial role of teacher. They stated that any type of behavior from teachers at any time can be considered as a model. Therefore, students will learn both positive and negative behaviors from their teachers. When students notice that what teachers are saying while teaching in the classroom is different from what they are actually expressing, that is, there is no coordination between their beliefs and actions, students will not pay attention to what they say, and they will not seek to act on them.

Regarding the role of language learners and their emotion in language teaching process, Tze et al. (2013) stated that discrepancy and inconsistency may also be seen between teachers’ expectations of language learners and the facilities that are actually provided to them. Language learners’ confidence in teachers’ leadership power will gradually diminish if teachers warn language learners that they are not allowed to leave the classroom before the bell rings, but in practice fails to implement the rule. They believed that language learners’ behavior is also influenced by teachers’ actions in the following cases: teachers emphasize the importance of reading a book, but they themselves do not read a single book. Teachers respect and value ethics, but are constantly confronted with anger and shouting at language learners on minor issues. Teachers emphasize that respect for the opinions and ideas of others, especially students, but they often ridicule language learners’ opinions that they find to be incorrect and stupid.

In all of these cases, language learners tend to engage in the behaviors and reactions that they actually display, rather than paying attention to what the teachers are expressing theoretically. Violent teachers with hostile behaviors have detrimental effects on language learners’ behavior, while kind teachers with non-aggressive behaviors can help their language learners’ social adjustment (Xie et al., 2021). Some of the signs of hostile behavior include exercising power, embarrassment, and insisting that students be obedient. For gentle and logical behaviors, teachers can also confirm language learners’ opinions; encourage them to participate in class discussions, express interest in students’ activities, and students’ sympathy and understanding. Students behave more consistently and gently in classes run by a kind and reasonable teacher, and the opposite is also true.

Wang G. et al. (2021) summarized that it should not be forgotten that teachers can have a tremendous impact on the classroom environment and thus on student behavior. Between teachers and students, the influence of students on teachers’ behavior should not be ignored, because this influence necessarily takes place in a two-way process. In most cases, the behavioral pattern and reactions (positive and negative) of teachers in the classroom have a significant relationship and coordination with the type of behaviors and reactions of students in the classroom. Similarly, Rigby and Ryan (2018) stated that teaching in the classroom takes the form of a two-way relationship in which students and teachers form an intertwined unit. For example, when students misbehave in the classroom, it affects teachers’ attitudes toward them, which in turn affects students’ attitudes toward their teachers. These effects play an essential role in raising and lowering the level of students’ boredom and learning.

## Conclusion and Implications

The following list describes the main findings of the literature and interpretations and important principles in creating good teacher-students’ relationships:

• After identifying their feelings, teachers should try to eliminate negative emotions such as: fear, guilt, suffering, emptiness, distrust, and enmity in a logical way and replace them with positive emotions such as: security, happiness, acceptance, trust in self, worth, love, and gratitude. They should apply the principle that I cannot relate to others unless I have a positive attitude and feeling toward them (the principle of having a positive attitude).
• Teachers must believe in the principle that human beings normally convey their feelings and emotions to students in the same way that their teachers convey their feelings and emotions to them (the principle of exchange equilibrium).
• Teachers must be able to express or stop the transmission of their emotions and feelings as best they can (principle of controlling emotions).
• Teachers should be able to describe their feelings and emotions using words, phrases, and gestures so that students understand their positive emotions well. Having a gentle and praiseworthy attitude toward students helps to achieve this (the principle of transmitting emotions and feelings).

The findings of the study showed that boredom and motivation are multidimensional aspects of language teaching and numerous factors such as educational tasks, classroom activities, educational environment, and learners’ individual differences might influence learners’ motivation and boredom; therefore, future studies are required to focus on these variables to find a feasible solution to increase learners’ motivation and decrease their boredom in any educational context.

## Author Contributions

The author confirms being the sole contributor of this work and has approved it for publication.

## Conflict of Interest

The author declares that the research was conducted in the absence of any commercial or financial relationships that could be construed as a potential conflict of interest.

## Publisher’s Note

All claims expressed in this article are solely those of the authors and do not necessarily represent those of their affiliated organizations, or those of the publisher, the editors and the reviewers. Any product that may be evaluated in this article, or claim that may be made by its manufacturer, is not guaranteed or endorsed by the publisher.
